# A Single Coronary Artery Originating from the Right Coronary Sinus with a Typical Course of the Right Coronary Artery and the Interarterial Course of the Left Main, Left Anterior Descending, and Left Circumflex as an Example of a Rare Case of High-Risk Coronary Anomaly

**DOI:** 10.3390/diagnostics12010167

**Published:** 2022-01-11

**Authors:** Paweł Gać, Aleksandra Żórawik, Rafał Poręba

**Affiliations:** 1 Centre for Diagnostic Imaging, 4th Military Hospital, Weigla 5, 50-981 Wroclaw, Poland; 2 Department of Population Health, Division of Environmental Health and Occupational Medicine, Wroclaw Medical University, Mikulicza-Radeckiego 7, 50-368 Wroclaw, Poland; aleksandra.zorawik@student.umw.edu.pl; 3 Department of Internal and Occupational Diseases, Hypertension and Clinical Oncology, Wroclaw Medical University, Borowska 213, 50-556 Wroclaw, Poland; rafal.poreba@umw.edu.pl

**Keywords:** computed tomography angiography, coronary anomaly, high-risk anomaly

## Abstract

In the typical course of the coronary arteries, the right coronary artery comes from the right coronary sinus and descends in the right atrioventricular groove. The left coronary artery trunk begins from the left coronary sinus. It crosses the pulmonary trunk and divides into left anterior descending and left circumflex arteries. Anatomical differences of the coronary arteries can be observed in 0.3–5.6% of the population. The interarterial course of coronary branches between the aorta and the pulmonary trunk is a malignant anomaly of the coronary arteries. Such abnormalities have been associated with an increased risk of sudden cardiac death. We present a rare case of coronary arteries anomaly involving the presence of a single right coronary artery and the interarterial course of its atypical branches documented by computed tomography angiography (CTA). In summary, the accurate assessment of the anatomical topography of coronary anomalies, possible in CTA, is necessary in the analysis of the risk of sudden cardiac death and its prevention.

## 1. Introduction

The presence of anatomical or morphological changes with a frequency > 1% of the general population is considered a variant of the norm. These are alternative, relatively atypical varieties that are seen in >1% of the same population. An anomaly is defined as changes that occur in <1% of the general population [1,2].

In the typical course of the coronary arteries, the right coronary artery comes from the right coronary sinus and descends in the right atrioventricular groove. In 50% of cases, its first branch is the conus branch, and the second branch is the sinoatrial node branch (but in 38% of individuals the sinoatrial node branch comes from the left coronary artery) [3]. In about 85% of population, the posterior descending branch originates from the right coronary artery (right dominant coronary supply) [3,4].

The left main coronary artery begins from the left coronary sinus. It crosses the pulmonary trunk and divides into left anterior descending (LAD) and left circumflex (LCx) arteries (but in about 37% of the population the left main coronary artery is divided into three: LAD, LCx, and intermediate branch) [3,5]. The LAD is located in the anterior interventricular groove and gives off diagonal branches. The LCx is located in the left atrioventricular groove and in about 7–8% gives off branches to the posterior wall of the right ventricle (left dominant coronary supply) [5,6]. Another 7–8% are cases when the posterior interventricular septum is supplied with blood by branches of the right coronary and circumflex arteries (codominance) [3].

The LAD travels in the anterior interventricular groove and gives off diagonal branches towards the anterolateral wall of the left ventricle. The LCx travels in the left atrioventricular groove and varies both in size and extent, depending upon the coronary dominance. It gives off one to three marginal branches supplying the free wall of the left ventricle [3].

Anatomical differences of the coronary arteries can be observed in 0.3–5.6% of the population [4,7,8]. Manifestations and pathophysiological mechanisms of this group of congenital disorders are highly variable [4]. Normal anatomical variants of the coronary arteries are a phenomenon of limited clinical significance, with no significant impact on the life of an individual. In contrast, in the group of coronary anomalies (which are generally rare and usually asymptomatic [9]) in addition to the mild variants (approximately 80% of anomalies are considered benign [8]) a group of lesions that potentially causes symptoms and poses a significant risk of sudden cardiac death can be distinguished (the remaining 20% [8]). It is the second leading cause of sudden cardiac death in young professional athletes (after hypertrophic cardiomyopathy) [3,10]. According to the Sudden Death Committee of the American Heart Association, coronary anomalies account for 19% of deaths in this group [1]. Interestingly, deaths due to coronary anomalies in the young general population are not that common. Drory et al. [11] investigated 162 cases of sudden deaths in a moderately active group of young people and found only 1 coronary anomaly. Burke et al. [12] compared sudden cardiac deaths in group of 14–40 years old individuals: coronary anomalies were found in 12% of sports-related sudden cardiac deaths vs. 1.2% in cases of non-athlete individuals’ deaths. According to Krasuski et al. [13] the risk of sudden death of sportsmen with coronary abnormalities is 79 times higher than in non-athlete person. Similar results from these and other studies suggest that some of coronary anomalies can be lethal only under certain circumstances, such as relationship with exercise. This is the reason why cases of sudden deaths mostly occur during (or right after) forceful physical activity [1].

As already mentioned, patients with coronary anomalies are mostly asymptomatic but in the group of symptomatic individuals the most frequent manifestations include atypical chest pain, dyspnea, exercise-related syncope/pre-syncope, arrhythmia, and left ventricular dysfunction [3].

Coronary anomalies can be classified into three traditional groups: anomaly of origination and course, anomaly of intrinsic coronary arterial anatomy, and anomaly of coronary termination [3,14], Table 1.

Most of the anomalies belong to the first group. The 3 main subcategories of group 1 include absent left main coronary artery, anomalous ostium outside of the aortic sinuses, and anomalous ostium at an improper sinus. Ropers et al. described 4 variants of the anomalous course of the left main coronary artery or the left anterior descending artery derived from the right Valsalva sinus: posterior or retroaortic course, interarterial or preaortic course, anterior or prepulmonic course, as well as septal or subpulmonic course [14]. There are also two less common subcategories: anomalous location of the coronary ostium in the aortic root and single coronary artery, which is one of the rarest anomalies [2,3].

Anomalies of intrinsic coronary anatomy include congenital ostial stenosis/atresia, coronary ectasia and aneurysm, subendocardial or intramural coronary course (myocardial bridging), duplicated arteries, and coronary crossing which is typically seen as a benign incidental finding on conventional or coronary computed tomography angiography [3].

Coronary artery fistulas belong to the third group. They are identified in about 0.15% of the population and can result in myocardial ischemia via a hemodynamic steal phenomenon [1,4].

In addition to anatomical criteria, the varieties of the coronary arteries can be classified according to their impact on the life of an individual. The benign course is not clinically significant. The malignant course is found to be a potential underlying cause of sudden cardiac death [10]. The most important prognostic factors are origination and proximal course of the variant coronary arteries [3]. The following categories are distinguished among hemodynamically significant abnormalities anomalies of origination with interarterial course; anomalous origin in the pulmonary artery; atresias; and congenital fistulas [3].

The most frequent type of hemodynamically significant malformation is the interarterial course of coronary artery with abnormal origin of the vessel from the opposite sinus of Valsalva—the pathway of coronary artery between the aorta and the pulmonary arterial trunk [3,10]. The right coronary artery originates from the left coronary sinus in 0.03% to 0.17% of population and in most cases has an interarterial course in its proximal pathway [3,14]. The left coronary trunk originates from the right coronary sinus in 0.09% to 0.11% of population. Three-quarters of these cases are related to the pathway between the aorta and the pulmonary arterial trunk [3,15].

At the same time, it is a malformation with the highest risk of sudden cardiac death [16]. This type of vascular malformation was found in 80% of autopsied athletes who were victims of coronary anomalies [17]. Interarterial course may result in constriction of anomalous vessel during intense physical activity. Additional risk factors constitute other anatomical conditions: crevice-like ostium, acute angle of take-off, or an intramural course of the anomalous vessel which results in proximal narrowing of shape [4,10].

Benign variants of coronary artery originating from opposite sinus represent all other courses, such as behind the aorta or in front of the pulmonary trunk [8].

One of the most severe anomalies is the anomalous origin of the coronary artery from the pulmonary artery. In most cases symptoms of this malformation are manifested in the first weeks of life, and 90% of untreated children die in the first year of life [2,3,16]. The most common variant is the Bland–White–Garland syndrome in which the right coronary artery originates from the aorta and left coronary trunk begins from the pulmonary artery [2,3].

Atresias may be caused by a congenital osteal stenosis caused by a membrane of fibrotic ridge [8]. Congenital osteal stenosis is not associated with atherosclerosis or other type of acquired disease. This type of malformation can cause significant narrowing of the coronary vessel.

In case of congenital fistula, the coronary artery ends in a low-pressure pulmonary vessel or in a cardiac chamber instead of gradual branching into capillaries in the myocardium. Small fistulas are often accidental findings; however, large fistulas lead to the steal phenomenon and inappropriate myocardial perfusion [8].

## 2. Case Report

We present a rare case of coronary arteries anomaly involving the presence of a single right coronary artery and the interarterial course of its atypical branches documented by computed tomography angiography (CTA).

The presented case concerns a 77-year-old woman admitted to an emergency cardiology ward due to the symptoms of unstable angina.

The patient reported in a history typical ailment of angina involving dyspnea and burning pain in the chest after physical effort (walking 100–200 m) and performed exercise stress testing was clinically positive. Physical examination revealed no symptoms at rest, HR approx. 70 bpm, BP 170/80 mmHg. Laboratory tests, except for hyperglycemia, showed no significant abnormalities.

The coronary angiography revealed a stenosis of 60–70% in the medial section of the LAD without any other significant stenosis in coronary arteries and the abnormal origin and course of coronary arteries. To visualize the course of the coronary arteries, CTA examination of the coronary arteries was performed additionally.

CTA examination was performed with a 384-layer CT device (Siemens Force) in the algorithm for the assessment of coronary arteries in the thickness layers of 3.0, 1.5, 1.0, and 0.6 mm. The presence of an anomaly of the coronary arteries was confirmed.

**Figure 1:**
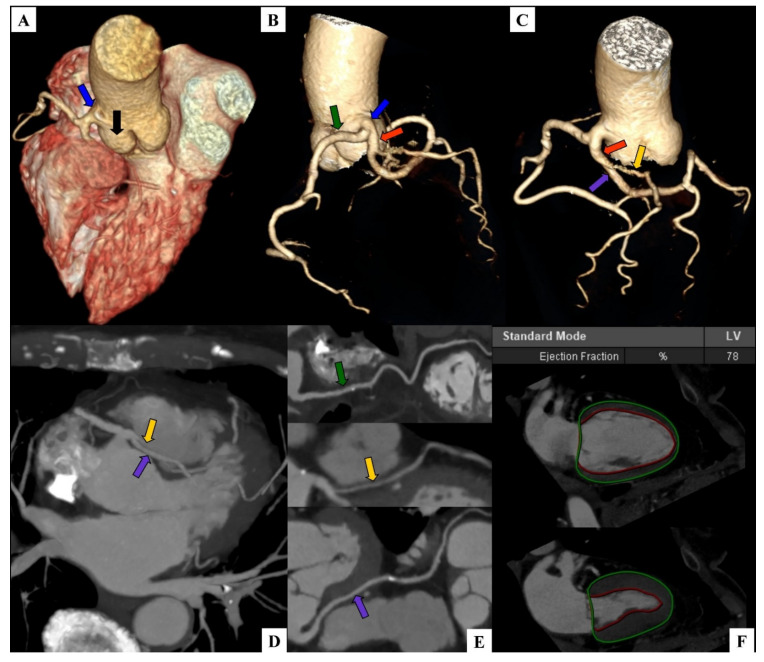
A single coronary artery originating from the right coronary sinus with a typical course of the right coronary artery and the interarterial course of the left main, left anterior descending, and left circumflex in coronary computed tomography angiography. On all panels, the arrows indicate: black—right coronary sinus, blue—single coronary artery, green—typical right coronary artery (RCA), orange—atypical left coronary artery (LCA), yellow—atypical left anterior descending artery (LAD), purple—atypical left circumflex artery (LCx). (**A**) Volume rendering technique (VRT). The origin of a single coronary artery from the right coronary sinus. (**B**) Volume rendering technique (VRT) with isolation of the vascular tree. Division of a single coronary artery into a typical RCA and an atypical LCA. (**C**) Volume rendering technique (VRT) with isolation of the vascular tree. Division of an atypical LCA into an atypical LAD and an atypical LCx. (**D**) Maximum intensity projection (MIP). Axial view. The interarterial course of LAD and LCx (**E**) curved planar reformation (CPR). Sequentially from the top: typical course of a right coronary artery, atypical course of a left anterior descending artery, atypical course of a left circumflex artery. (**F**) Left ventricular functional assessment. Left ventricular ejection fraction (EF)—78%.

The presence of a single coronary artery originating from the right coronary sinus was revealed (Figure 1A). Approximately 4–5 mm from the ostium this single coronary was divided into 2 large branches: a branch with a course typical for the dominant right coronary artery (RCA) and a branch with a circulation supplying corresponding to the left coronary artery (LCA) (Figure 1B).

Its course shows a short atypical left main coronary artery (LM) with an interarterial course dividing about 7–8 mm into 2 branches with the nomenclature corresponding to the left anterior descending artery (LAD) and the left circumflex artery (LCx) (Figure 1C). Atypical LCA branches had an interarterial course (Figure 1D). A narrow proximal atypical segment of the LAD ran in the adipose tissue between the aortic bulb and the pulmonary artery. The next LAD sections showed morphology and the course of a typical LAD, located in the anterior interventricular groove (Figure 1E).

The proximal segment of atypical LCx was wider than the corresponding section of LAD and located lower and deeper—within the myocardium of the ventricular septum and the anterior wall of the left ventricle on about 25–30 mm. After exiting the myocardium, it gave a strong first marginal branch (OM1), tended upward, and further sections showed a course and morphology typical for the typical LCx (Figure 1E).

Based on the CTA of the coronary arteries, a diagnosis of a single coronary artery originating from the right coronary sinus with a typical course of the right coronary artery and the interarterial course of the left main, left anterior descending artery, and left circumflex artery as a rare case of high-risk coronary anomaly was given.

Additionally, the coronary calcium score was measured, and it was 133.4 (LAD 0.2, LCx 73.8, RCA 59.3). Moreover, atherosclerotic plaques of various morphotic types causing short-segment stenosis of the coronary arteries (of the degree as in the previously performed coronary angiography) were revealed.

There were no calcifications in the mitral and tricuspid aortic valves, no thickening and/or calcification of the pericardium, and no pathological pericardial fluid volume. Automatically volumetrically estimated by the CT post-processing application left ventricular ejection fraction was normal and equals 78% (norm range: 57–79%) (Figure 1F).

The significance of LAD stenosis was objectified with a dobutamine test, which was echocardiographically negative. The patient was consulted with the heart team. Due to the overall clinical situation, it was decided not to qualify the patient for interventional treatment. It was decided to intensify pharmacological treatment. Regular cardiological control was recommended, with the necessity to re-qualify for interventional treatment if the patient’s clinical condition changes. Due to the center’s experience, qualification for CABG would be considered first in interventional treatment. Due to comorbidities, the following pharmacotherapy was recommended: clopidogrel 75 mg, acetylsalicylic acid 75 mg, perindopril 5 mg, metoprolol 25 mg, amlodipine 5 mg, atorvastatin 40 mg, trimetazidine 35 mg, isosorbide 10 mg, metformin 1000 mg, empagliflozin 10 mg, and thiamazole 5 mg.

## 3. Discussion

Congenital coronary artery anomalies are rare findings in normal population. Prevalence of RCA or LCA originating from opposite Valsalva sinus varies, depending on performed study and type of detection method. According to Angelini et al., the total prevalence rate is 1.07% (with use of coronary angiography), divided into 2 groups: 0.92% RCA from the left sinus of Valsalva and 0.15% LCA from the right [17]. A single coronary artery (SCA) originating from the right or left sinus constituted 3.3% of all coronary anomalies in the performed study [18]. As an isolated finding the occurrence of anomalous coronary artery from the opposite sinus (ACAOS) is extremely low (0.024–0.044%) [18,19,20,21,22,23,24,25]. In approximately 17–19% of the reported cases (according to other sources, up to 40%), it is associated with other congenital anomalies (persistent truncus arteriosus, pulmonary atresia, tetralogy of Fallot) [18,19,20]. It should be noted that the data on the incidence of coronary anomalies are at risk of high selection bias. Most individuals in the population do not undergo coronary angiography or other imaging studies aimed at assessing the anatomy of the coronary circulation.

Desmet et al. [24] reported that the first case of single coronary artery was described in 1716 by Thebesius. About 45 cases of single coronary artery had been documented until 1950 [26]. In 1979, it was presented very useful classification of anomalies which includes single coronary arteries [19,20]. In this classification system ‘III’ means: single coronary artery, after leaving the right coronary sinus of Valsalva LAD and LCx arise separately from proximal part of the artery [19,23].

Mechanisms of the pathogenesis of the development of single coronary artery are not exactly known [20]. Symptoms of anomalous coronary artery from the opposite sinus (ACAOS) can present very similarly to symptoms of coronary artery disease, especially in cases with interarterial course [25]. This can be seen in this case, where the patient was admitted to a cardiology ward due to the symptoms of unstable angina and reported in a history typical ailment of angina involving dyspnea and burning pain in the chest after physical effort.

The case where a single coronary artery originates from the right coronary sinus is very rare. Ozyurtlu et al. described a case single coronary artery originating from right sinus Valsalva, dividing into right coronary artery (RCA) and the left main coronary artery which was giving two branches as left anterior descending artery (LAD) and left circumflex (LCx) after traversing the base of heart. The patient reported typical chest pain [23]. Pergola et al. described two similar cases. Both patients reported epigastric pain with nausea, vomiting, vertigo, and weakness [26].

Cases of a single coronary artery originating from the left coronary sinus are also rare. Gholoobi et al. reported an extremely rare variation of a single coronary artery originating from the left sinus of Valsalva with retroaortic course [27]. The patient presented with unstable angina. Previously, the case of a left-sided ACAOS with the interarterial course was reported by Gravina et al. [28]. The patient was complaining of exertion chest discomfort. Both variations may lead to myocardial ischemia and sudden cardiac arrest which are usually associated with its course between the aorta and main pulmonary artery [20]. But generally, right-sided anomalous coronary artery from the opposite sinus (RCA from the left sinus) is considered to be less malignant than other coronary artery anomalies, such as a left-sided ACAOS (LCA from the right sinus) [17]. Described mortality rate is 57% for left-sided ACAOS and 25% for right-sided ACAOS [29]. In other words, the case where a single coronary artery originates from the right coronary sinus and then divides into the arteries corresponding to RCA and LCA (LCA with interarterial course) is characterized by a higher risk than when a single coronary artery originates from the left coronary sinus (RCA with interarterial course) [30]. However, there are of course symptomatic cases of right-sided ACAOS. For instance, a case was described of a patient diagnosed with RCA originating from the left sinus of Valsalvas, presenting symptoms of unstable angina [17].

In the treatment of patients with an anomalous origin of coronary artery from opposite sinus with interarterial course, it is essential to carefully stratify the risk, considering the risk of sudden cardiac death on the one hand and the risk of planned interventions on the other hand. In patients with a high risk of sudden cardiac death, surgical treatment should be considered: coronary unroofing, coronary reimplantation, or CABG. The assessment of the consequences of the diagnosed anomaly, especially the verification of myocardial ischemia, is important in the risk assessment. Due to the existing risk of complications from interventional treatment, non-invasive treatment should be considered in patients without documented myocardial ischemia [31]. Coronary unroofing can result in coronary commissural damage and aortic insufficiency. A complication of coronary reimplantation may be stenosis of the new coronary artery ostium. After CABG, the bypass may clot rapidly due to the competitive flow in the coronary anomaly, which may only be slightly reduced at rest [31]. Taking into account the above-mentioned conditions, in the described case, due to the general clinical condition (especially no signs of myocardial ischemia in the dobutamine test), it was decided to use non-invasive treatment. At the same time, the prospect of a possible qualification to CABG in the event of progression of the degree of intermediate stenosis in the middle segment of the LAD was indicated.

## 4. Conclusions

Accurate assessment of the anatomical topography of coronary anomalies, possible in computed tomography angiography, is essential in the analysis of the risk of sudden cardiac death and its prevention.

## Figures and Tables

**Table 1:** Coronary anomalies classification.

Anomalies of Origination and Course	Intrinsic Anomalies	Termination Anomalies
Absence of left main coronary artery Coronary ostium outside the aortic coronary sinus: pulmonary artery, left ventricle, right ventricle, ascending or transverse aorta, etc. Coronary ostium in improper coronary sinus: right coronary artery originating from the left coronary sinus, left anterior descending and/or circumflex arteries originating from the right coronary sinus, with proximal course anomaly (posterior or retroaortic course, interarterial or preaortic course, anterior or prepulmonic course and septal or subpulmonic course) Anomalous location of the coronary ostium in the aortic root: high, low, commissural Single coronary artery	Atresia or congenital ostial stenosis, ectasia or aneurysm, hypoplasia, agenesis, etc. Subendocardial or intramural course (myocardial bridge) Split right coronary artery and anterior descending artery, anomalous origin of the posterior descending artery or first septal branch	Fistulas Anomalies of arteriolar/capillary branching
